# Prognostic follow-up of high-grade squamous intraepithelial lesions of the cervix near the conization margin: A retrospective cohort study

**DOI:** 10.1371/journal.pone.0331184

**Published:** 2025-09-12

**Authors:** Dandan Chen, Huamin Wu, Feifeng Huang, Kailiang Tan

**Affiliations:** Department of Obstetrics and Gynecology, Maternal and Child Health Hospital of Guangxi Zhuang Autonomous Region, Nanning, China; Mater Olbia Hospital, ITALY

## Abstract

**Objective:**

This study explored the prognosis of high-grade squamous intraepithelial lesion (HSIL) patients with negative margins, investigating the impact of different distances between lesions and incisal margins in conization specimens.

**Methods:**

This retrospective cohort study included 240 HSIL patients. Patients with negative incisal margins were divided into 3 groups according to the distance between the lesion and the incisal margin in the conization tissue. Group 1 consisted of a distance of <1 mm (n = 23), Group 2 of 1–3 mm (n = 15), and Group 3 of >3 mm (n = 202). For patients with lesions close to the incisal margin (≤3 cm), the decision between total hysterectomy and clinical observation was made based on patient preference following detailed counseling of disease characteristics and prognosis. Thinprep cytologic test (TCT) and HPV testing were performed during follow-up at 6 and 12 months after the operation.

**Results:**

No significant difference in HPV and TCT positive rate was observed among the three groups at 6 months and 12 months after the operation (P = 0.561, 0.561 and P = 0.324, 0.268). In the group with a distance shorter than 3 mm, no difference in HPV positive rate was found between the total hysterectomy and observation groups (P = 0.480, 0.737). Additionally, no difference in HPV positive rate was observed between patients who underwent total hysterectomy compared to clinical observation in groups 1 and 2 (P = 0.565, 0.692; P = 0.758, 0.593). Stratified analysis revealed that HPV positive rates at 6 months and 12 months had no statistical significance with any factor.

**Conclusion:**

Different distances between conization tissue lesions and incisal edges have no direct impact on the prognosis of HSIL patients with negative conization biopsy tissues; excessive hysterectomy is not recommended in patients (≤3 mm) close to incisal edges.

## Introduction

Cervical cancer is one of the most common cancers in women. According to the latest data, the number of new cases of cervical cancer worldwide reached 661,000 in 2022, with a death toll of 348,000 [1]. Notably, the condition affects a progressively younger population [2]. Cervical squamous intraepithelial lesion (SIL) is closely related to cervical cancer. In 2020, the World Health Organization (WHO) proposed a global strategy to accelerate the elimination of cervical cancer, aiming to reach the goal of “90-70-90” by 2030. Specifically, the goals include 90% of girls being vaccinated against HPV (Human Papillomavirus) before age 15, 70% of women being screened by highly effective test methods before age 35 and 45, and 90% of women diagnosed with precancerous lesions being treated according to standardized protocols. These measures emphasize the importance of refined management of SIL to prevent unnecessary diagnosis and treatment [3,4].

However, the indication of hysterectomy for high-grade squamous intraepithelial lesions (HSIL) remains controversial [5,6]. Positive results from conization specimens (especially positive results from inner margins) are a high risk factor for residual lesions and recurrence [7]. Therefore, patients with HSIL who exhibit a positive incisal margin following conization should be closely monitored, particularly those with a low-risk incisal margin, before considering additional surgical intervention. Conversely, factors supporting total hysterectomy include histologically confirmed residual or recurrent HSIL with difficulty performing re-conization, age > 50 years with positive internal margin [8,9]. Nonetheless, some other studies revealed that human papillomavirus (HPV) persistence after conization was an independent risk factor for predicting residual or recurrent lesions after conization [10,11], presenting a higher accuracy than that of the incisal margin status [12]. Alder S et al. prospectively followed up 991 patients undergoing cervical conization for cervical intraepithelial neoplasia (CIN)2/3. The results revealed that persistent HPV infection after surgery increased the risk of residual lesions and recurrence after conization in patients with positive incisal margins [13]. While considering only the positive margin, the possibility of residual lesions, persistent HPV infection, and recurrence in the negative margin may be overlooked. In conization specimens in which the lesion is close to the incisal margin, residual microlesions indiscernible to the naked eye may still be present..

The 2024 NCCN Clinical Practice Guidelines for Cervical Cancer recommend a negative margin for microinvasive carcinoma, and the 2023 NCCN guidelines have been changed from at least 3 mm to 1 mm [14]. However, the definition of positive margin of cervical intraepithelial neoplasia has not been standardized. Some early studies considered CIN lesions to have positive margins when they appeared within 1 mm of the margin [15]. In patients with negative incisal margins but lesions in close proximity to the incisal margin, clinical concern about recurrence may warrant total hysterectomy. Still, whether this scheme reduces the risk of recurrence remains undetermined.

Cervical conization techniques mainly include cold knife conization (CKC) and loop electrosurgical excision procedure (LEEP). Clinically, the two methods are widely used, showing similar curative effects [16,17]. However, some studies suggest that CKC can provide original specimens without electrothermal burns at the incisal margin, which does not affect pathological diagnosis and has a low recurrence rate after surgery [18].

This retrospective cohort study investigated the prognosis of HSIL patients with negative conization tissue margins at different distances from the incisal margin, providing a reference for the selection of individualized treatment options for patients.

## Materials and methods

### Patients

This retrospective cohort study included patients with HSIL who received conization surgery in the Gynecology Department of Maternal and Child Health Hospital of Guangxi Zhuang Autonomous Region from February 2023 to December 2024. Inclusion criteria: 1. Pathologically confirmed cervical intraepithelial lesion; 2. All patients were treated with cold knife conization (CKC) in our hospital; 3. Conization specimen with negative incisal margin confirmed by pathology in our hospital after operation; 4. Generally good condition, stable vital signs, normal liver and kidney function, and complete case and follow-up treatment available. Exclusion criteria: 1. Irregular treatment in other hospitals; 2. CKC treatment was not performed in our hospital; 3. Unstable vital signs; 4. Abnormal liver and kidney function, cardiopulmonary function, and coagulation function; 5. Postoperative diagnosis of invasive cervical cancer, adenocarcinoma in situ, or positive incisal margin. Before treatment, patients were informed in detail about the treatment process, potential risks, and effects of each regimen. After full consideration, patients decided on the treatment regimen and signed informed consent. This study was approved by the Medical Ethics Committee of Maternal and Child Health Hospital of Guangxi Zhuang Autonomous Region, and all patients in this study signed written informed consent. [No: Z-A20230347]. Data were accessed for research purposes on May 31, 2025.

### Cervical conization

To eliminate possible bias in the outcome of the study due to the surgical procedure, only patients who underwent CKC were analyzed in this study. The procedure steps included administration of intravenous anesthesia, routine disinfection, sterile draping, and CKC of the cervix. The cervix was stained with a compound iodine solution to delineate lesion margins and assess for residual abnormal epithelium. The depth was determined according to biopsy pathology, lesion scope, and the patient’s fertility requirements. The resection width exceeded 0.3 cm, and hemostasis was secured using electrocoagulation. An absorbable suture was utilized, and the cervical canal was packed with iodoform gauze to ensure hemostasis within 24h after surgery. Then, a marking suture was placed at the bottom of the cone at 12 o’clock, and the specimen was sent for pathological analysis. The distance between the lesion and the incisal margin of the negative conization biopsy tissue was measured. According to the distance, the lesions were divided into various groups: Group 1 distance <1mm, Group 2 distance 1–3 mm, and Group 3 distance >3 mm. For patients with lesions close to the incisal margin (≤3 cm), the patients were informed of the disease characteristics and prognosis, and were allowed to decide between total hysterectomy or clinical observation.

### Negative margin criteria

Absence of HSIL at any of the three incisal edges (medial incisal edge, lateral incisal edge, and basal incisal edge) in the first conization specimen, the incisal edge was deemed negative.

### Observation and follow-up

Age, number of pregnancies, parity, delivery method, menopausal status, HPV status before conization, thinprep cytologic test (TCT) status before conization, pathology before conization, pathology after conization, width of conization, and height of conization were collected. Cervical HPV and TCT were followed up 6 and 12 months after the operation.

### Statistics

SPSS 26.0 software was used for data analysis. Normal distribution was expressed as mean ± standard deviation, and analysis of variance was used for comparison between groups. Specifically, M (P25-P75) was used to compare variables not conforming to a normal distribution, and the Kruskal–Wallis H test was used for comparison between groups. The X^2^ test and independent sample t-test were used for comparison between groups, applying continuity correction or Fisher’s exact test where appropriate. Stratified analysis was performed with R programming language (v 4.1.4.2). In this study, P < 0.05 was statistically significant.

## Results

### Characteristics of patients

**Fig 1:**
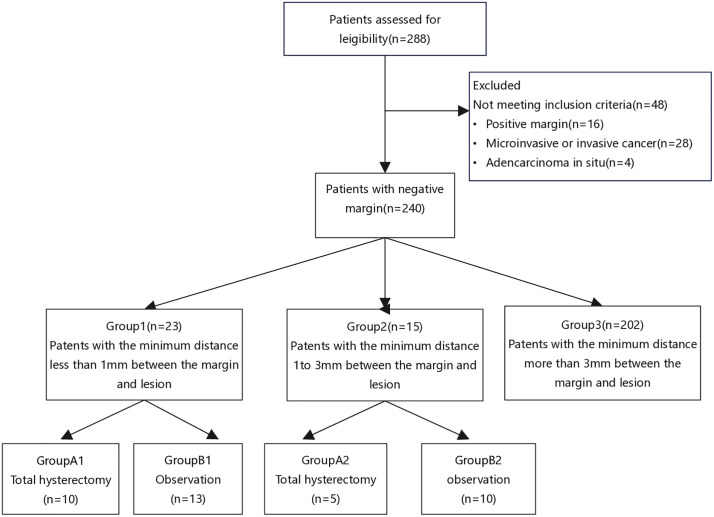
Flow diagram of the study.

A total of 288 patients with HSIL were included in this study. Specifically, 48 patients who did not meet the criteria were excluded, and 240 patients were finally included in the study, comprising 23 patients in Group 1, 15 patients in Group 2, and 202 patients in Group 3 (Fig 1). No statistically significant differences were found among the three groups in terms of age, number of pregnancies, number of births, delivery method, menopausal status, HPV status before conization, TCT status before conization, pathology before conization, pathology after conization. In Group 3, 6 patients became pregnant after the operation. The complications included cervical insufficiency (1 case), bleeding (2 cases), and cervical adhesion (2 cases), as displayed in Table 1.

**Table 1:** Characteristics of patients.

Variables	Group1 (n = 23)	Group2 (n = 15)	Group3 (n = 202)	P
Age	41.0 ± 8.73	43.3 ± 6.73	40.0 ± 10.07	0.189
Number of pregnancies	3(0-7)	3(1-7)	3(0-9)	0.442
Number of births	2(0-4)	2(1-3)	2(0-7)	0.593
Delivery method				
Nulliparous	3(13.0%)	(0)0%	30(14.9%)	0.312
Natural labor	16(69.6%)	10(67.7%)	140(69.3%)	
Caesarean section	4(17.4%)	5(33.3%)	32(15.8%)	
Menopausal status				
Premenopausal	18(78.2%)	11(73.3%)	178(88.1%)	0.139
Postmenopausal	5(21.7%)	4(26.7%)	24(11.9%)	
HPV status before conization				
High risk	8(34.8%)	3(20%)	93(46.0%)	0.177
Non-High risk	15(65.2%)	11(73.3%)	105(52.0%)	
Negative	0(0%)	1(6.7%)	4(2.0%)	
HPV status before conization				
Positive	20(87.0%)	11(73.3%)	135(66.8%)	0.123
Negative	3(13.0%)	4(26.7%)	67(33.2%)	
Pathology before conization				
CIN2	16(69.6%)	7(46.7%)	134(66.3%)	0.275
CIN3	7(30.4%)	8(53.3%)	68(33.7%)	
Pathology after conization				
CIN2	8(34.8%)	5(33.3%)	108(53.5%)	0.093
CIN3	15(65.2%)	10(67.7%)	94(46.5%)	

Values are presented mean ± SD, median(minimum–maximum) and (%).

Group 1: patients with the minimum distance less than 1 mm between the margin and lesion; Group 2: patients with the minimum distance 1–3 mm between the margin and lesion; Group 3: patients with the minimum distance more than 3 mm between the margin and lesion.

### Follow-up results at 6 and 12 months in three groups of patients

No statistical difference in cervical TCT results was found among the three groups at 6 months and 12 months of follow-up (P = 0.561). Notably, 6 months after the operation, HPV-positivity was observed in 1 case (4.3%) in Group 1, 3 cases (20.0%) in Group 2, and 23 cases (11.4%) in Group 3, showing no significant difference across the three groups (P = 0.324). Moreover, 12 months after operation, HPV positivity was observed in 2 cases (8.7%) in Group 1, 4 cases (26.7%) in Group 2, and 27 cases (13.7%) in Group 3, demonstrating no significant difference (P 0.268), as listed in Table 2.

**Table 2:** Follow-up results at 6 and 12 months in three groups of patients.

Variables	Group1 (n = 23)	Group2 (n = 15)	Group3 (n = 202)	P
Abnormal results of TCT 6 months after conization	(0)0%	(0)0%	6(3.0%)	0.561
Abnormal results of TCT 12 months after conization	(0)0%	(0)0%	6(3.0%)	0.561
HPV positivity rate at 6 months follow-up	1(4.3%)	3(20%)	23(11.4%)	0.324
HPV positivity rate at 12-month follow-up	2(8.7%)	4(26.7%)	27(13.4%)	0.268

Values are presented as n (%).

Group 1: patients with the minimum distance less than 1 mm between the margin and lesion; Group 2: patients with the minimum distance 1–3 mm between the margin and lesion; Group 3: patients with the minimum distance more than 3 mm between the margin and lesion.

### Follow-up results of different therapeutic schedules for patients with lesions near the incisal margin

Patients with partial conization tissue lesions near the incisal margin (distance ≤3 mm) were concerned about disease progression and tended to opt for total hysterectomy. A total of 38 patients showed lesions near the incisal margin (distance ≤3 mm), among which 15 patients underwent total hysterectomy (group A) and 23 patients were observed. TCT was negative for 6 and 12 months after surgery. In addition, 6 months after operation, HPV positivity was found in 1 patient in group A and 3 patients in group B, showing no significant difference (P = 0.480). Similarly, 12 months after operation, HPV positivity was observed in 2 cases in group A and 4 cases in group B, indicating no significant difference (P = 0.737).This is shown in Table 3.

**Table 3:** Follow-up results of different therapeutic schedules for patients with lesions near the incisal margin.

Variables	Group A (n = 15)	Group B (n = 23)	P
Abnormal results of TCT 6 months after conization	(0)0%	(0)0%	1.0
Abnormal results of TCT 12 months after conization	(0)0%	(0)0%	1.0
HPV positivity rate at 6 months follow-up	1(6.7%)	3(13.0%)	0.480
HPV positivity rate at 12-month follow-up	2(13.3%)	4(17.4%)	0.737

Values are presented as n (%).

Group A: Patients with total hysterectomy after conization with minimum distance ≤3 mm between incisal margin and lesion. Group B: Patients with minimum distance ≤3 mm between incisal margin and lesion and observed after conization.

### Follow-up results of different therapeutic schedules in patients with lesions at a distance less than 1 cm|

Total hysterectomy (group A1) was performed in 10 cases, and observation (group B1) in 13 cases. TCT was negative 6 months and 12 months after the operation. No patient in group A1 and 1 patient in group B1 was HPV-positive at 6 months after operation (P = 0.565). HPV positivity was found in 1 case in group A1 and 1 case in group B1 at 12 months after the operation (P = 0.0.692). About 10% (1/10) of patients had residual lesions after total hysterectomy, which were CIN2. The rest were negative. This is shown in Table 4.

**Table 4:** Follow-up results of different therapeutic schedules in patients with lesions at a distance less than 1 cm.

Variables	GroupA1 (n = 10)	Group B1 (n = 13)	P
Abnormal results of TCT 6 months after conization	(0)0%	(0)0%	1.0
Abnormal results of TCT 12 months after conization	(0)0%	(0)0%	1.0
HPV positivity rate at 6 months follow-up	(0)0%	1(7.7%)	0.565
HPV positivity rate at 12-month follow-up	1(10%)	1(7.7%)	0.692

Values are presented as n (%).

Group A1: Patients with a minimum distance between the incisal margin and lesion of less than 1 mm and total hysterectomy after conization. Group B1: Patients with a minimum distance between the incisal margin and lesion of less than 1 mm and observed after conization.

### Follow-up results of different therapeutic schedules in patients with lesions at a distance of 1–3 cm

Total hysterectomy (group A2) was performed in 5 cases, and observation (group B2) in 10 cases. TCT was negative 6 months and 12 months after the operation. HPV positivity was observed in 1 case in group A2 and 2 cases in group B2 at 6 months after operation, showing no statistical difference (P = 0.758). HPV positivity was found in 1 patient in group A2 and 3 patients in group B2 at 12 months after operation, revealing no statistical difference (P = 0.593). About 40% (2/5) of the patients had residual lesions after hysterectomy. One case of CIN2 and one case of CIN3 were recorded, while the rest were negative.This is shown in Table 5.

**Table 5:** Follow-up results of different therapeutic schedules in patients with lesions at a distance of 1- 3 cm.

Variables	Group A2 (n = 5)	Group B2 (n = 10)	P
Abnormal results of TCT 6 months after conization	(0)0%	(0)0%	1.0
Abnormal results of TCT 12 months after conization	(0)0%	(0)0%	1.0
HPV positivity rate at 6 months follow-up	1(20%)	2(20%)	0.758
HPV positivity rate at 12-month follow-up	1(20%)	3(30%)	0.593

Values are presented as n (%).

Group A2: Patients with a minimum distance of 1–3 mm from the margin to the lesion who underwent total hysterectomy after conization. Group B2: Patients with a minimum distance of 1–3 mm from the margin to the lesion and observed after conization.

### Stratified analysis of factors correlated with HPV positivity

**Fig 2:**
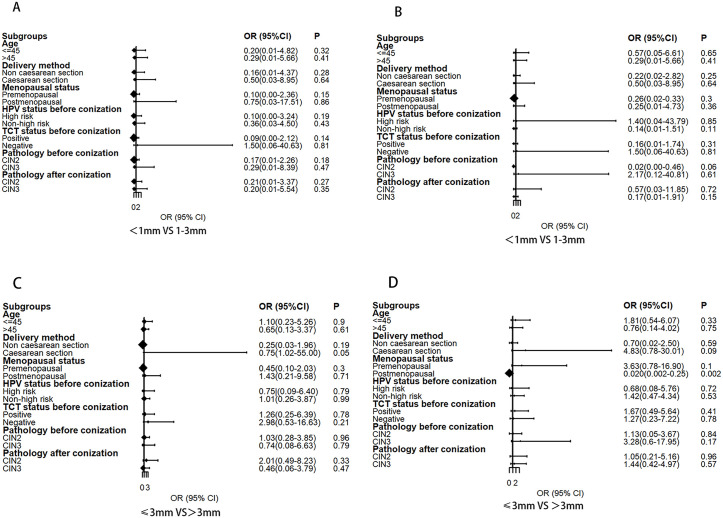
Stratified analysis of factors correlated with HPV positivity. (A) HPV- positive rate at 6 months after operation between patients with <1mm vs 1-3 mm. (B) HPV- positive rate at 12months after operation between patients with <1mm vs 1-3 mm. (C) HPV- positive rate at 6 months after operation between patients with ≤3 mm vs > 3mm. (D) HPV- positive rate at 12 months after operation between patients with ≤3 mm vs > 3mm.

Age, delivery method, menopausal status, HPV status before conization, TCT status before conization, pathology before conization, and pathology after conization were analyzed. The results revealed no statistical difference in HPV positivity rate at 6 months and 12 months after operation among any of the factors, as displayed in Fig 2.

## Discussion

With the development of cancer screening in China, a growing number of patients have received early diagnosis and early treatment. CKC removes lesions while preserving cervical anatomy and female fertility. CKC has been widely used in the treatment of precancerous lesions of the cervix. Despite adequate treatment of HSIL, some women still relapse within 2 years. Multiple studies have confirmed that a positive margin after cervical conization is a risk factor for residual lesions, persistent HPV infection, and recurrence [8,9,12]. The incidence of persistence of lesions after cervicectomy in HSIL patients was 18% in patients with positive margins and 4.3% in those with negative margins [19]. Fan et al. found that the recurrence rate of negative margins after cervical conization was only 8.1% [20]. The 2023 NCCN guidelines suggest that a lesion less than 1 mm from the incisal margin is positive [14]. Recent studies suggested that a positive incisal margin should be followed up [21]. Nonetheless, some patients opt for total hysterectomy to minimize the risk of cancer. This study redefines the positive incisal margin. On the one hand, whether there is any difference in prognosis between patients with lesions close to incisal margin (≤3 mm) and those further from incisal margin (>3mm); on the other hand, this article discusses whether patients with lesions within 1 mm and 3 mm from incisal margin can benefit from total hysterectomy after conization. Currently, no relevant reports have been published. In this study, 240 patients were followed up for 12 months. All patients underwent colposcopy and were pathologically negative. The residual rate of HPV in patients with negative margins at 6 months was 11.3% (27/240), and that in patients with negative margins at 12 months was 13.8% (33/240), indicating that the residual rate was low, which was consistent with clinical conditions [22].

Lin et al. showed that the recurrence rate of CIN without residual HPV and negative margins after LEEP was only 0.5%. However, the recurrence rate of CIN patients with negative margins but residual HPV was 18%. The HPV residual rate of CIN patients with negative margins was 22.7% [22], 

suggesting the importance of cervical HPV infection, exceeding that of the margin state. Nevertheless, whether the recurrence of patients with negative incisal margins is related to the proximity of the lesion to the incisal margin remains poorly understood. Burak Giray et al. recruited 74 patients with a negative cone incisal margin and assigned them to three groups (incisal margin distance <2 mm, 2–5 mm, > 5 mm) in 2019 [23]. The results revealed no statistical difference in the proportion of cytological abnormal results between the groups at the last 6 months and 12 months (6 months: P = 0.33; 12 months: P = 0.80). HPV persistence rates also showed no difference between groups during follow-up (6 and 12 months) (P = 0.93 and P = 0.89). This suggests that the distance of the lesion from the incisal margin (even if <2 mm) does not affect the risk of recurrence if the incisal margin is negative. The present study divided patients into three groups (margin distance <1 mm, 1–3 mm, > 3mm). The results indicated no statistical difference between the 6-month and 12-month TCT groups (6 months: P = 0.560;12 months: P = 0.560); similarly, no difference in HPV positive rates was found (6 months: P = 0.324;12 months: P = 0.141). These findings indicate that in the negative margin state, the distance of the lesion from the margin had no significant effect on postoperative recurrence in HSIL patients. These results are consistent with the only studies available.

The incidence of HSIL increases every year, with the age of affected individuals tending to be younger. Cervical conization removes a part of cervical tissue and leads to cervical shortening, which increases the risk of premature delivery, low birth weight, and premature rupture of membranes in the future [24]. Clinicians pay increasing attention to the impact of cervical conization on future pregnancy outcomes. Efforts are being made to completely remove the lesion without affecting future pregnancy. Cui et al. retrospectively studied 176 patients with CIN2 and revealed that CKC increased the risk of premature delivery (14.4%), premature rupture of membranes (13.6%), and cesarean section (37.5%) compared with healthy pregnant women (p < 0.05) [25]. However, Mehmet et al. showed that patients with CKC were more likely to have premature delivery when their cervical canal length was less than 31 mm (P < 0.001), and premature rupture of membranes was more likely when their cervical canal length was less than 32 mm (P = 0.007) [26]. At present, there are no guidelines indicating the minimum safe distance between the lesion and the excision margin in cases with negative margins. The study showed no pregnancies in Group 1 and Group 2, and 6 pregnancies in Group 3. Among these, cervical incompetence showed an incidence of 16.7% (1/6). Post-conization complications included bleeding in 1.0% (2/202) and intrauterine adhesions in 0.5% (1/202) of patients. Guo, HJ et al. reported that the premature birth rate was significantly higher in patients who underwent CKC compared to LEEP (38.88% vs. 20.5%), OR = 2.455 (1.007–5.985) [27]. Shin, JW et al. found that CKC treatment had a significantly lower incidence of non-negative surgical margins than LEEP treatment (14.3% vs. 52.6%; p < 0.05) in patients>45 years of age, suggesting that LEEP is a better option for women who want to preserve fertility; in contrast, CKC is a better option for women who do not have fertility requirements, especially those>45 years of age [28]. However, the study had limitations, such as not including healthy female patients as controls. Further research is needed to optimize the surgical scope by investigating the effect of different incisal margin distances on obstetric complications such as premature delivery.

Meta-analysis conducted by Ghaem-Maghami et al. found that residual lesions and recurrence occurred in 4% of women with negative margins after conization. The incidence of postoperative HSIL was 18% in patients with incomplete resection, whereas it was only 3% in patients with complete resection [19]. Some scholars have shown that the persistence/recurrence of HSIL after resection treatment may be due to the persistence of HPV infection after resection [29]. Therefore, scholars believe that total hysterectomy after conization can significantly reduce the occurrence of residual lesions, thereby reducing the recurrence rate. Total hysterectomy does reduce the risk of recurrence to some extent, but the quality of life and physical and mental health of patients are affected to some extent, resulting in excessive treatment, causing physical and psychological harm to patients. A positive margin is not an independent risk factor for lesion persistence, especially considering that programmed electrocoagulation of LEEP can clear lesions with uncut margins. Therefore, total hysterectomy is not warranted in all patients with positive margins. In this study, TCT was negative at 6 months and 12 months after total hysterectomy (≤3 mm) compared with the observation group, and no difference in HPV positive rate was found between the total hysterectomy and observation groups (P = 0.480, 0.737). In Group 1, TCT was negative at 6 months and 12 months after total hysterectomy compared with the observation group, and the HPV positive rate showed no significant difference at 6 months and 12 months after operation (P = 0.565, P = 0.692). Among the patients who underwent total hysterectomy, postoperative pathology revealed residual lesions in only one patient. In Group 2, total hysterectomy was negative at 6 months and 12 months after the operation compared with the observation group, and the HPV-positive rate demonstrated no statistical difference at 6 months and 12 months after the operation (P = 0.758, P = 0.593). Only two cases of total hysterectomy patients had residual uterine lesions in postoperative pathology. The study revealed no statistically significant difference in prognosis between the total hysterectomy and observation groups in patients with lesions near the incisal margin. Patients with lesions near the incisal margin are not recommended to undergo total hysterectomy.

Increasing age was associated with a higher HPV positive rate after conization. Gustafson et al. followed up 100 elderly women who had undergone diagnostic cervical resection with a median age of 67.4 years for 2.9 years. The results showed that 70% of elderly women were HPV negative and 30% were HPV positive. The HPV-positive rate was higher in women older than 65 years [30]. Stratified analysis of this study revealed no statistical difference in HPV positive rates between premenopausal and postmenopausal women. The age of the patients included in the present study is younger than that in the literature. Vaccination of HSIL patients may reduce the incidence of different HPV-associated lesions [31,32]. Therefore, women are recommended to actively take cervical vaccines to prevent cervical HPV incidence, rather than total hysterectomy.

In addition to positive margin, recurrence of HSIL was associated with age, preoperative endocervical curettage (ECC) pathological grade, immunosuppressive state, inflammatory markers, and HR-HPV persistent infection [33,34]. Zivadinovic et al. found that age and persistent HR-HPV infection are risk factors for the recurrence of postoperative lesions, especially in patients with negative margins [35]. HPV E6/E7 mRNA can mediate the degradation of tumor suppressor genes and interfere with the regulation of the cell cycle. In the early stage of HPV infection, the expression level of HPV E6/E7 mRNA is low, with a low risk of cervical carcinogenesis. When the expression level of HPV E6/E7 mRNA increases, it can activate telomerase expression, which leads to tumor cell proliferation and increases the risk of carcinogenesis. Therefore, the risk of cervical carcinogenesis can be effectively assessed by detecting the expression level of HPV E6/E7 mRNA. Zappacosta et al. reported that all mRNA-positive results were associated with a recurrent CIN. Among mRNA-negative women, about 54% were related to liquid-based cytology (LBC)/DNA positivity detected during a 6-month visit. HPV-mRNA detection combined with TCT detection achieved higher specificity and positive predictive value at follow-up after conization. Such management could further reduce follow-up frequency [36]. Moreover, E6/E7 mRNA HPV detection has a high detection efficiency for HSIL patients [37], supporting the notion that patients with negative margins do not need to undergo total hysterectomy. HPV-mRNA detection combined with TCT detection can be used for follow-up screening after conization to identify patients with postoperative recurrence or residual high risk, and targeted treatment can be carried out.

In conclusion, this study provides an important basis for clinical practice. Different distances between conization tissue lesions and incisal edges have no direct impact on the prognosis of HSIL patients with negative conization biopsy tissues; hysterectomy is not recommended in patients (≤3 mm).

Nevertheless, the limitations of the present study should be acknowledged. The included sample size of cases with lesions near the incisal margin was small, resulting in a large difference in sample size; moreover, the follow-up time was short, so a longer follow-up is required to evaluate the long-term prognosis, complications, and quality of life of patients. Future research should investigate the influence of different distances between lesions and incisal margin on obstetric complications, such as hemorrhage, premature delivery, low birth weight, and perinatal mortality, in patients with a negative incisal margin. This approach promotes individualized and refined management while mitigating overdiagnosis and overtreatment.

## Supporting information

S1 File Dataset. (XLSX)
